# Making free public healthcare attractive: optimizing health equity funds in Cambodia

**DOI:** 10.1186/s12939-018-0803-3

**Published:** 2018-06-25

**Authors:** Bart Jacobs, Ashish Bajracharya, Jyotirmoy Saha, Chhorvann Chhea, Ben Bellows, Steffen Flessa, Adelio Fernandes Antunes

**Affiliations:** 1 Social Health Protection Programme, Deutsche Gesellschaft für Internationale Zusammenarbeit (GiZ), c/o NIPH, No.2, Street 289, Khan Toul Kork, P.O. Box 1238, Phnom Penh, Cambodia; 2 Population Council, Phnom Penh, Cambodia; 3 Population Council, Dhaka, Bangladesh; 4 National Institute of Public Health, Phnom Penh, Cambodia; 5 Population Council, Lusaka, Zambia; 6 Department of General Business Administration and Health Care Management, University of Greifswald, Greifswald, Germany; 7 SOCIEUX + Expertise on Social Protection, Labour and Employment, Brussels, Belgium

**Keywords:** Access, Health financing, User fees, Healthcare utilization, Exemption mechanism, Poverty, Equity

## Abstract

**Background:**

Following the introduction of user fees in Cambodia, Health Equity Funds (HEF) were developed to enable poor people access to public health services by paying public health providers on their behalf, including non-medical costs for hospitalised beneficiaries (HEFB). The national scheme covers 3.1 million pre-identified HEFB. Uptake of benefits, however, has been mixed and a substantial proportion of poor people still initiate care at private facilities where they incur considerable out-of-pocket costs. We examine the benefits of additional interventions compared to existing stand-alone HEF scenarios in stimulating care seeking at public health facilities among eligible poor people.

**Methods:**

We report on three configurations of HEF and their ability to attract HEFB to initiate care at public health facilities and their degree of financial risk protection: HEF covering only hospital services (HoHEF), HEF covering health centre and hospital services (CHEF), and Integrated Social Health Protection Scheme (iSHPS) that allowed non-HEFB community members to enrol in HEF. The iSHPS also used vouchers for selected health services, pay-for-performance for quantity and quality of care, and interventions aimed at increasing health providers’ degree of accountability. A cross sectional survey collected information from 1636 matched HEFB households in two health districts with iSHPS and two other health districts without iSHPS. Respondents were stratified according to the three HEF configurations for the descriptive analysis.

**Results:**

The findings indicated that the proportion of HEFB who sought care first from public health providers in iSHPS areas was 55.7%, significantly higher than the 39.5% in the areas having HEF with health centres (CHEF) and 13.4% in the areas having HEF with hospital services only (HoHEF). The overall costs (out-of-pocket and transport) associated with the illness episode were lowest for cases residing within iSHPS sites, US$10.4, and highest in areas where health centres were not included in the package (HoHEF), US$20.7. Such costs were US$19.5 at HEF with health centres (CHEF).

**Conclusions:**

The findings suggest that HEF encompassing health centre and hospital services and complemented by additional interventions are better than stand-alone HEF in attracting sick HEFB to public health facilities and lowering out-of-pocket expenses associated with healthcare seeking.

## Background

Over the last decade, more evidence has demonstrated that the cost of health care constitutes a major barrier to timely access of services, especially for poor people and vulnerable populations, and represents a key barrier to address if progress is to be made moving toward universal health coverage [1, 2]. In response, many governments of low-income countries have abolished user fees for all public health services or key health services such as tuberculosis treatment and institutional deliveries while also creating exemptions for specific population groups such as poor people, children and pregnant women [2–4].

Results of such initiatives to enable free health care at the point of delivery to date are mixed. In Uganda, where user fees were abolished for public health services, the private sector remained the main source of curative care with consequent increased out-of-pocket expenses for health [5, 6]. In Zambia, catastrophic health expenses remained high amongst poor people despite entitlements for free health care [1]. In other countries, user fee abolition or provision of subsidised access led to an initial increase in utilisation of public health services though the growth rate was not sustained [7, 8]. One study that employed control sites found no increase in utilisation amongst poor people following their entitlement to free care [9].

The counterintuitive failure of user fee abolition initiatives to universally improve uptake of public health services and reduce out-of-pocket expenses amongst intended beneficiaries occurs when the initiative does not offer alternative financial resources for facilities losing user fee revenue, which then undermines the quality of care [2]. As a result of this poor quality, households seek care in the private-for-profit sector [6] where services are more expensive [10, 11].

Cambodia introduced user fees in the public health sector in the late 1990s as a means to collect more revenue and to stimulate delivery of services by staff members. Unlike experiences from other countries, utilisation of public sector health services increased although the poor saw their financial access decreased [12, 13]. In order to safeguard the positive effects of user fees on staff performance while ensuring access to health care for the poor, so called Health Equity Funds (HEF) were established. Health Equity Funds are third-party arrangements that pay public health facilities the user fees for services rendered to eligible poor [14]. Eligibility for HEF is assessed by the Ministry of Planning through a nationwide community-based exercise, the IDPoor Programme, conducted every 3 years, using proxy means testing. Those missed during this targeting exercise can be considered for fee waivers when reporting at the hospital during the so-called post-identification exercise.

Health equity fund coverage has expanded over time and evidence suggests that, on average, beneficiary households reduce their out-of-pocket spending on healthcare and seek care less frequently in the private sector. However, a substantial proportion of HEF beneficiaries still initiate healthcare seeking at private health providers where they incur considerable out-of-pocket expenses [15, 16]. One study found that HEF decreased out-of-pocket expenses for health services amongst the entitled poor people but did not increase their utilisation of public health services [17]. More recently a study comparing health care utilisation and out-of-pocket expenses for health services amongst poor people with and without HEF benefits (e.g. IDPoor cardholders) found that HEF did increase utilisation of public health facilities. Health equity fund beneficiaries (HEFB) who used their IDPoor card to access public health services during their illness spent much less than those who didn’t make use of their card or those without entitlements [18].

To reduce the financial hardship due to health expenses, it is thus important to ensure that HEFB initiate care seeking at public health facilities, so they will presumably spend less money. Here we report on the effects of three HEF configurations on financial access to public health facilities by HEF beneficiaries.

### Integrated social health protection scheme

Health Equity Funds emerged in the early 2000s as a pragmatic response to balance positive and negative effects associated with user fees in the public health sector. Initially a variety of approaches existed [19]. With increased donor and government interest and consequent bigger external funding, management of the HEF became increasingly institutionalised [20]. Geographical expansion of HEF occurred incrementally and by the end of 2015 enrolled all public health facilities in the country. In many places, HEF co-existed with other health financing interventions aiming at improving access to (selected) health care services such as internal contractual arrangements, performance-based financing, and voucher schemes [21].

To improve service delivery and utilisation at public health facilities, the Ministry of Health piloted the Integrated Social Health Protection Scheme (iSHPS) in the rural province of Kampong Thom, central Cambodia, with support of the Cambodian-German Social Health Protection Programme (SHPP). The scheme combined HEF with the ability for non-holders of ID-Poor cards to enrol by paying a small fee that entitled them to access the same medical services as the HEF eligible poor people for free at the point of delivery. The inclusion of non-HEFB in iSHPS and the rebranding of the HEF scheme to iSHPS aimed at destigmatising holders of IDPoor cards. The implementation of this strategy was accompanied by awareness raising activities to stimulate voluntary enrolment in the iSHPS by non-holders of IDPoor cards.

**Fig. 1:**
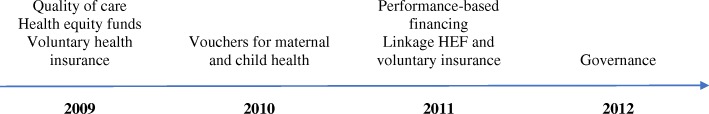
Chronological implementation of interventions at iSHPS sites

A selected set of underutilised maternal and child health services were promoted amongst the population and reimbursed through vouchers. Health centres were reimbursed for services delivered under the iSHPS on a pay-for-performance basis that combined output payments based on annually negotiated targets and adjusted by objective quality and client satisfaction scores. Targets were set for each health centre using achievements of previous years as a benchmark. The iSHPS intervention area also benefited from a limited set of interventions under the SHPP that aimed at improving health systems governance structures to increase health providers’ degree of accountability and responsiveness. Investments were also made in technical and structural quality of health services (Fig. 1).

This paper reports results of a post-intervention evaluation of the iSHPS, which examines its effectiveness to attract eligible poor beneficiaries to the public health sector to receive free care as well as their degree of financial risk protection compared to alternative configurations of the HEF that were implemented in Cambodia. Specific attention is paid to the initiation of care seeking and associated costs in the public sector among three different configurations of HEF:

1. *iSHPS* in which HEF coverage is extended to households not identified as poor by the IDPoor programme for a small membership fee;
2. *Standard HEF* in which HEF coverage is only available at a hospital to eligible poor households (henceforth abbreviated HoHEF); and
3. *Comprehensive HEF* in which HEF coverage is available at both the health centre level and the hospital level to eligible poor households (henceforth abbreviated CHEF).

In doing so, the study examines the additional benefits of the add-on interventions of the iSHPS in stimulating care seeking at public health facilities among the eligible poor compared to existing stand-alone HEF scenarios.

## Methods

### Study design and data

This study utilizes data from a cross-sectional household survey and employs a post-intervention evaluation design with control groups to evaluate the impact of the iSHPS on healthcare seeking and related out-of-pocket expenditures among eligible poor compared to stand-alone HEF configurations operating in Cambodia.

Data were collected from four operational health districts (OD) between October 2013 and February 2014. Two ODs, Kampong Thom and Stong in Kampong Thom province, where the iSHPS pilot had been implemented, were selected as intervention areas. Similarly, two ODs where the HEF scheme had been implemented without participation in the pilot iSHPS were chosen as control areas, Maung Russey OD, Battambang province, and Chamka Leu OD, Kampong Cham province, based on their similarity to the intervention ODs along the following parameters: geographical location, population density, and percent of the population eligible for HEF. The two control ODs also were chosen to represent two distinct configurations of HEF implementation. Maung Russey OD has a Comprehensive HEF (CHEF) with coverage at all of its health centers. In Chamka Leu OD, many health centers had not been covered by HEF at the time of the survey, representing a Hospital Only HEF (HoHEF) configuration with coverage only at the hospital level.

The study respondents comprised a sample of men and women aged between 18 and 59 years. Respondents were interviewed on their health seeking behavior and health-related and socio-demographic characteristics. To achieve a sufficient sample size to compare changes on various health related variables between intervention and control groups, an appropriately powered minimum detectable effect sample size calculation was performed. Assumptions included an alpha of 0.05, power of 0.8 and a design effect factor of 1. The parameter and related assumption was the proportions of HEFB seeking care at public health facilities when ill in the month preceding interview (40% at intervention vs. 29% at control sites).

Open Epi for sample size calculation was used to estimate the sample size. To detect an 11-percentage point difference in care seeking at intervention and control ODs, taking into account loss to follow up (20%) and refusal rate (20%), the number of interviewees needed per OD was 434. Control and interventions sites each comprised two ODs and using a 1:1 ratio, 868 respondents each from intervention and control sites, of the appropriate age, had to be approached for interview for a total required sample size of 1736. The final analytical sample collected for the study included a total of 1636 respondents: 767 respondents from iSHPS (intervention areas) and 869 respondents from the control ODs.

Selection of individuals in the intervention areas was done using a list of HEF member households that were identified as eligible through the IDPoor exercise. The number of such households to be selected from each OD was determined proportionate to the size of total number of IDPoor households in each OD and chosen through random selection in each intervention OD. Following selection of the households, respective health centers and village of residence were identified.

At the control areas a list of all health centers was generated from the most recent records and matches were sought for these facilities with health centres at iSHPS sites, based on selected parameters, including outpatient consultations, antenatal care visits, deliveries, number of staff members and catchment area population. Following pairing of health centres, an identical number of villages from the catchment areas of each of the matched health centers was selected randomly. In each selected control village, a list of IDPoor households was utilized to randomly select households into the study. At both intervention and control sites a household was replaced by another IDPoor household if the sampled household was no longer present in the village of origin under which they were listed or when nobody was at home when approached for interview. None of the present eligible household representatives objected to interview.

### Tools

The survey implemented two tools. The first tool, a household roster, asked questions on key demographic variables for every member of the household. This questionnaire was answered by an adult member of the household, preferably female aged 18–59 years, recruited as the main respondent for the study and concerned issues related to socio-economic status. The second tool was an individual questionnaire that was administered to the same main respondent. This questionnaire covered topics about the main respondent’s perceived health status, utilization, source of healthcare services and healthcare expenditures. This tool also collected, from the main respondent, information regarding other members of the household who had episodes of sickness, particularly on their healthcare utilization and expenditures.

### Statistical analysis

Respondents and sick household members were stratified according to HEF configuration and analysed regarding the relationship between HEF configurations and kind of providers consulted for the concerned illness episode and associated OOP spending. The unit of analysis was the household with individuals, including the main respondent, clustered within households. Health providers were differentiated as public (health centre or hospital), private qualified (pharmacies, private clinics) and non-qualified informal providers (drug shops, traditional healers, and market vendors hereinafter termed non-medical providers). Only illness episodes during the month preceding interview were considered. Costs involved only direct medical (fees and medicine) and non-medical (transport) out-of-pocket expenses incurred by the patients while care seeking for the concerned illness episode. Payments in Khmer Riels (KHR) were converted to US$ at KHR4,000 to US$1. Although information on costs was collected in a time period covering parts of two calendar years, no discount rate was applied to adjust these figures. Means were used as averages for cost data [22].

Differences in the distribution of the different HEF configurations were calculated and Chi- square tests were conducted to test for statistical significance of differences between categorical variables and t-tests were performed for continuous variables, determined at the 5% level (*p* < 0.05), after adjusting for cluster effects, with individuals clustered within households. The cost and distance variables were tested for normality with the Shapiro-Wilk test and found to violate the assumption of normality which could not be improved by transformation. Therefore, the non-parametric Kruskal-Wallis test with cluster adjustment was used to test for statistical significance of overall group differences [23]. To test for pair-wise differences between HEF configuration pairs, the Dunn’s test was subsequently performed with the Bonferroni correction, as the appropriate nonparametric pairwise multiple comparison procedure following a Kruskal-Wallis test [24].

### Definitions used

- Direct medical costs: out-of-pocket payments for health services
- Direct non-medical costs: out-of-pocket payments for transport
- Total cost per treatment: sum of direct medical and non-medical cost for concerned treatment
- Overall cost: sum of total costs for first and second treatment

### Ethical considerations

This research was approved by two Ethical Review Boards: the Population Council Institutional Review Board, New York, and the Cambodian National Ethics Review Committee for Health Research. All interviewees were read the consent statement and requested to sign or thumbprint when agreeing with the interview.

## Results

### Sample characteristics

A total of 1636 households were approached and had one adult member interviewed (Table 1). There was a considerable difference for the gender of these respondents whereby men accounted for 28.6% at HoHEF sites, nearly double the proportion found at iSHPS, 14.7%, while the figure was 20.9% in CHEF areas. The proportion of households with at least one sick household member was especially low for the CHEF at 57.0% versus 81.7–83.8% for the other configurations. While nearly all sick cases reportedly sought health care, the lowest figure was reported for CHEF, 92.2%. The average age of sick cases seeking health care was lowest at iSHPS, 22.8 years and highest for CHEF, 29.0 years. Children made up 25.5% of those seeking care at iSHPS, more than 5 percentage points higher than at control sites. The proportion of sick men or women of reproductive age making up the sample of care seekers was statistically similar across the sites.

**Table 1:** Main characteristics of the respondents for the HEF configurations

	HEF configuration			*p*-value^¶^
	HoHEF* N (%)	CHEF* N (%)	iSHPS** N (%)	
Number of health centres	4	9	27	
Number of respondents	262	607	767	
Gender respondent				
Male	75 (28.6)	127 (20.9)	113 (14.7)	< 0.001
Female	187 (71.4)	480 (79.1)	654 (85.3)	
Had ≥1 sick member	214 (81.7)	346 (57.0)	643(83.8)	< 0.001
Total sick persons	414	486	1182	
Gender of sick person				
Male	200 (48.3)	197 (40.7)	538 (45.6)	NS
Female	214 (51.7)	287 (59.3)	642 (54.4)	
Was sick and sought care	411 (99.3)	450 (92.2)	1153 (97.6)	< 0.001
Mean age of sick seeking care in years. Of which	26.3	29.0	22.8	< 0.001
children aged ≤5 years	82 (20.0)	87 (19.3)	294 (25.5)	0.008
women of reproductive age	75 (18.3)	102 (22.7)	258 (22.4)	NS

*HoHEF* hospital only HEF, *CHEF* comprehensive HEF, *iSHPS* integrated social health protection scheme, *NS* not significant; *control sites; **intervention site; ^¶^*p*-value derived from Chi-square test for categorical variable and t-test for continuous variable after adjusting for cluster

### First treatment

When sick and seeking care, half the cases of iSHPS (48.7%) reportedly did so at health centres, considerably higher than the proportions observed at CHEF, 29.0%, and especially HoHEF, 8.3% (Table 2). The difference between initiating care seeking at health centres between iSHPS and CHEF patients was highly significant (*p* < 0.001). The respective difference for care seeking at public hospitals was small, 7.0 and 10.5%, but still significant (*p* < 0.001). This was not the case for the difference between HoHEF and iSHPS. The total proportion consulting first public health providers in iSHPS areas was 55.7% significantly higher (*p* < 0.001) than the 39.5% observed at CHEF and 13.4% at HoHEF (*p* < 0.001 for comparison with iSHPS).

**Table 2:** Care seeking and associated costs at first provider (those who were sick)

	HoHEF N (%) *N* = 411	CHEF N (%) *N* = 450	iSHPS N (%) *N* = 1153	HoHEF vs CHEF	CHEF vs iSHPS	HoHEF vs iSHPS
Sought care at				Cluster adjusted Chi square < 0.001		
Health centre	34 (8.3)	130 (29.0)	559 (48.7)			
Public hospital	21 (5.1)	47 (10.5)	80 (7.0)			
Private facility	209 (50.8)	161 (36.0)	337 (29.3)			
Non-medical	147 (35.8)	110 (24.6)	172 (15.0)			
Total who went public	*55 (13.4)*	*177 (39.5)*	*639 (55.7)*			
Use of IDPoor card				Cluster adjusted Chi square < 0.001		
Health centre	19 (55.8)	121 (93.0)	422 (75.5)			
Hospital	9 (42.8)	29 (61.7)	40 (50.0)			
Any public facility	*28 (50.9)*	*150 (84.7)*	*462 (72.3)*			
Distance to provider in km [SD]				*p*-value*		
Health centre	1.7 [2.5]	5.4 [7.9]	3.4 [4.1]	< 0.001	0.007	0.001
Public hospital	16.8 [14.5]	35.3[21.9]	19.9 [23.3]	0.001	0.001	NS
Private facility	3.1 [9.5]	9.7 [12.3]	4.7 [9.0]	< 0.001	< 0.001	0.001
Non-medical	1.1 [1.1]	2.5 [5.3]	1.4 [2.8]	NS	NS	NS
Average per facility	*2.8 [8.1]*	*8.9 [14.2]*	*4.5 [9.1]*	< 0.001	0.001	< 0.001
Direct medical cost in US$ [SD]						
Health centre	1.6 [5.4]	3.1 [32.9]	0.08 [0.9]	< 0.001	NS	< 0.001
Public hospital	27.4 [45.3]	25.0 [60.3]	16.9 [57.7]	NS	NS	NS
Private facility	32.1 [64.0]	30.4 [46.6]	20.5 [33.3]	0.05	0.001	NS
Non-medical	3.3 [4.7]	6.4 [16.5]	3.4 [6.8]	NS	NS	NS
Average per patient	*19.1 [48.9]*	*15.9 [40.9]*	*7.7 [25.4]*	< 0.001	< 0.001	< 0.001
Direct non-medical cost in US$ [SD]						
Health centre	0.3 [0.4]	0.9 [1.5]	0.3 [1.6]	NS	< 0.001	NS
Public hospital	4.7 [4.8]	11.4 [15.4]	5.4 [7.1]	NS	NS	NS
Private facility	0.6 [1.4]	2.6 [6.4]	1.1 [2.5]	< 0.001	0.001	0.014
Non-medical	0.14 [0.3]	1.2 [7.0]	0.14 [0.4]	NS	NS	NS
Average per patient	*0.6 [1.8]*	*2.7 [7.8]*	*0.9 [2.9]*	< 0.001	< 0.001	1.0
Average total cost in US$ [SD]	19.7 [49.2]	18.6 [64.3]	8.6 [26.3]	0.014	< 0.001	< 0.001
Initiates care at public facilities	13.4	12.6	3.1	NS	< 0.001	0.001
Initiates care at private facilities	20.6	19.5	15.5	NS	NS	NS

*NS* not significant, *SD* standard deviation; * Bonferroni–Dunn post hoc test for multiple comparison after cluster adjusted Kruskal Wallis test

Sick cases from CHEF areas consulting public providers were significantly more likely to use their IDPoor card entitlements, 84.7%, than their iSHPS counterparts, 72.3% (*p* < 0.001). The lowest use of IDPoor Card was at HoHEF, 50.9%. All those using their IDPoor card when consulting public health providers did not pay for their health care. On average CHEF cases resided the furthest from the health providers they consulted, 8.9 km versus 4.5 km at iSHPS sites and 2.8 km for HoHEF cases (all differences *p* < 0.001). These differences were significantly different for distances to health centres, the public hospital (except for HoHEF vs. iSHPS), and private facilities.

The average direct medical cost for first treatment was lowest for those under the iSHPS, less than half the amount observed at CHEF, and two and a half times less than at HoHEF. This low cost for iSHPS cases appears partly due to lower charges at health centres and private facilities compared to control sites. The average direct medical cost for paying patients was US$0.08 at health centres in iSHPS sites versus US$1.6–3.1 in control sites although not significantly different between CHEF and iSHPS sites. There were also differences in such costs for those consulting private facilities, US$20.5 for such cases at iSHPS, compared with US$30.4 for CHEF and US$32.1 for HoHEF. This was only significantly different at CHEF vs. iSHPS facilities.

Because of the longer distances to travel, direct non-medical costs for CHEF cases was significantly more, US$2.7, than for other HEF configurations, US$0.6–0.9.

The total cost for the first treatment amounted to US$8.6 for iSHPS cases, about half the amount spent at CHEF and HoHEF. Those initiating care seeking at public health providers spent less than the ones doing so in the private sector. The respective figure, however, was by far the lowest in the iSHPS sites, up to a third and a quarter of the amounts observed at other sites.

### Second treatment

As seen in Tables 3, 21.5% of iSHPS patients reportedly went for a second treatment compared to < 15% of cases from the control sites. Many of the iSHPS (40.3%) and CHEF (36.4%) went to public health providers, although at HoHEF (45.1%) and iSHPS sites (46.8%) most went to private qualified providers while at control sites a considerable proportion (34.9 to 37.3%) went to non-medical providers.

**Table 3:** Second treatment and associated costs

	HoHEF N (%) N = 411	CHEF N (%) N = 450	iSHPS N (%) N = 1153	HoHEF vs CHEH	CHEF vs iSHPS	HoHEF vs iSHPS
				Cluster adjusted Chi square = 0.001		
Went for 2nd treatment	51 (12.4)	66 (14.7)	248 (21.5)			
Those who initiated care at public facility	7 (12.7)	32 (18.1)	146 (23.0)			
at private provider	29 (13.9)	14 (8.7)	74 (22.0)			
at non-medical provider	15 (10.2)	20 (18.2)	28 (16.3)			
Sought care for 2nd treatment at				Cluster adjusted Chi square = 0.001		
Health centre	3 (5.9)	15 (22.7)	72 (29.0)			
Public hospital	6 (11.8)	9 (13.6)	28 (11.3)			
Private facility	23 (45.1)	19 (28.8)	116 (46.8)			
Non-Medical	19 (37.3)	23 (34.9)	32 (12.9)			
>Proportion going to a public facility	*(17.6)*	*(36.4)*	*(40.3)*			
Direct medical cost in US$ [SD]				*p*-value*		
Health centre	0.5 [0.3]	0.1 [0.04]	0.2 [1.8]	< 0.001	NS	< 0.001
Public hospital	16.9 [33.7]	10.6 [26.2]	3.7 [16.8]	NS	NS	0.026
Private facility	10.1 [10.2]	10.4 [8.4]	13.9 [15.7]	NS	NS	NS
Non-Medical	2.7 [2.2]	1.8 [2.5]	3.5 [6.6]	NS	NS	NS
*Average per patient who sought 2nd treatment*	*7.5 [13.8]*	*5.0 [10.9]*	*7.4 [13.7]*	0.039	0.024	NS
Direct non-medical costs in US$ [SD]						
Health centre	0.3 [0.3]	0.8 [1.7]	0.3 [0.85]	NS	NS	NS
Public hospital	3.6 [3.0]	4.6 [4.1]	4.3 [4.6]	NS	NS	NS
Private facility	0.9 [1.7]	1.2 [1.3]	1.0 [1.7]	NS	NS	NS
Non-Medical	0.3 [0.91]	0 [0]	0.5 [2.2]	NS	NS	NS
*Average per patient who sought 2nd treatment*	*1.0 [1.9]*	*1.1 [2.2]*	*1.1 [2.4]*	NSS	NS	NS
Total cost 2nd treatment per patient who sought care in US$ [SD]	8.5 [14.9]	6.1 [11.7]	8.5 [14.4]	NS	NS	NS
Overall cost per patient who sought care in US$	20.7	19.5	10.4	NS	< 0.001	< 0.001
Of which treatment costs (% of total)	20.0 (96.6)	16.6 (85.1)	9.3 (89.4)			
Of which transport costs (% of total)	0.7 (3.4)	2.9 (14.9)	1.2 (10.6)			

*NS* not significant, *SD* standard deviation: * Bonferroni–Dunn post hoc test for multiple comparison after cluster adjusted Kruskal Wallis test

Direct medical costs for the second treatment were significantly lowest at CHEF sites in comparison with the other sites, $5.0, while such costs were similar at iSHPS and HoHEF, US$7.4 and US$7.5 respectively. With similar amounts for average transport costs at the three sites, the average total costs for cases who sought a second treatment were statistically similar but lowest at CHEF sites, US$6.1, compared to US$8.5 at the other sites.

### Overall costs

Overall costs associated with the illness episode were significantly lowest for cases residing within iSHPS sites, US$10.4, and highest in areas where health centres were not included in the package, US$20.7. Such costs were US$19.5 at CHEF. For the latter, direct non-medical costs made up 14.9% of overall costs while this was only 3.4% for HoHEF. At iSHPS sites transport costs made up 10.6%.

## Discussion

It has been argued previously that multiple interventions may be required to improve access to health care because of the numerous barriers that poor patients encounter [21]. As such, each additional intervention may assist in overcoming a specific access barrier. This argument appears reinforced by findings from this study, which indicated that 56% of HEFB residing in districts with iSHPS initiated care at public health facilities, higher than the 40% observed at Comprehensive HEF and much higher than the 13% for Hospital Only HEF. Care seeking for HEFB under the iSHPS was also associated with the lowest direct costs, the main objective of Health Equity Funds.

Inclusion of health centres as primary-level health care facilities contributed greatly to initiating care seeking at public health facilities. This is shown by the fact that only 8% of sick cases in HoHEF initiated care at health centres compared with 29% at CHEF sites and 49% for iSHPS. The difference in care seeking at health centres between the latter two HEF configurations suggests that factors in addition to health centre inclusion are at play since IDPoor card use was highest at CHEF health centres. Knowledge about entitlements associated with HEF was identified as an important factor to have beneficiaries effectively using associated services [18, 25].

Although we did not assess the influence of each intervention in additional to the HEF in the iSHPS area, their combined effects may have positively influenced care seeking at public health facilities. The governance aspects of the SHPP focussed on increasing accountability of health providers towards the public through their direct engagement using existing structures such as Commune Councils and Health Centre Managements Committees. Because of these governance activities, health providers increasingly interact with the public and are thus better known to them. Improved case finding for tuberculosis in Phnom Penh was ascribed to better communication by health providers with the community that resulted in enhanced confidence of patients to consult them [26]. Trust in public health providers was also found to be an important determinant for mothers to timely consult public health care providers for children with suspected dengue infection [27]. While Health Centre Management Committees should have been established at all such facilities in the country, external support likely improved their functioning as observed elsewhere [28]. Such community engagement also aids in improving quality and delivery of health services [29, 30]. However, the governance interventions were only implemented at about two thirds of health centres suggesting that additional factors influenced the findings.

At iSHPS sites, the activities were also complemented by pay-for-performance and a voucher scheme to stimulate delivery of –mainly preventive- health services. As such primary-level health care facility staff were paid quarterly bonuses based on community feedback through quarterly client satisfaction surveys as well as service delivery frequency, which improved interaction of the health care providers with the community. This is important as preventive health services are largely delivered during outreach sessions, thereby bringing the health providers to the villages and requiring collaboration of community representatives with organising delivery of these services. While outreach sessions occur nationwide, the pay-for-performance scheme may have improved such collaboration to increase coverage of target populations. Hence these activities may have induced an additional degree of accountability amongst public health providers as well as increased familiarity amongst community members. Children were more represented amongst iSHPS cases seeking care than at control sites, which suggests increased familiarity. It is likely that the voucher scheme that targeted mothers and children also contributed to increased interactions between these population groups and public health care providers.

The fact that significantly more cases in CHEF areas initiated care at the public hospital compared with iSHPS cases suggest that perceived quality of care may also affect choice of public health provider. This is underscored by the fact that cases in CHEF areas travelled nearly double the distance, 35.3 km, than those residing in iSHPS, 19.9 km. It is the more remarkable as transport is not reimbursed for HEF beneficiaries who bypass the health centre. Contrary, quality of care at iSHPS health centres may have been perceived as good.

Excluding health centres from the HEF benefit package may have wider ramifications on use of hospital services as indicated by the fact that only 5% of beneficiaries from such areas initiated care at the hospital even though the facility represented the only source of free care. Unlike health centres, contact with the hospital and its staff members is rare due to the low incidence of hospitalisation whereby HEF beneficiaries may refrain from accessing such facilities due to unfamiliarity with staff members. As experience from neighbouring Thailand suggests it is also necessary to improve the district health system that includes health centers and hospitals and not only the social health protection scheme to enable access to public health services [31]. It is, however, commendable to see that at HoHEF areas 56% of cases consulting health centres received fee waivers despite the fact that the facility is not reimbursed by the HEF. The tendency of health centres to provide such fee waivers, contrary to the practices by public hospitals, has been documented earlier [32].

Initiating care at public health facilities greatly reduced the total cost for the first treatment, in line with the HEF objectives. Those consulting public health providers for the first treatment spent on average US$3.1 (vs. US$15.5 at private providers) under the iSPHS, US$12.6 (vs. US$19.5) with CHEF and US$13.4 (vs. US$20.6) for HoHEF. iSHPS cases tended to initiate care seeking at health centres but a quarter of them did not use their IDPoor while only half of them consulting hospitals did so, significantly fewer than CHEF cases. User fees at iSHPS facilities, however, appeared much lower than at control sites. It could be that public health facility fees were lower at iSHPS because of the higher proportion of children for whom fees are set at rates lower than for adults. The average age of patients was also lowest at iSHPS sites, suggesting that age of the may have influenced care seeking decisions.

The fees charged by qualified private providers were also lowest at iSHPS but cases initiating care in the private sector had five times more total costs than their counterparts who went to public providers. This magnitude was less for such cases in control sites. The lower prices observed at iSHPS sites amongst private providers may result from the governance activities of the iSHPS as “dual practice” is common amongst public health providers [33] and because of increased exposure to the community it is likely that they are more inclined to align their fees with those prevailing at the public sector. It has been observed earlier [12] that the private health sector in rural areas tend to adjust their fees to the prices at the public sector so low fees in the public sector may benefit the wider population.

A substantial proportion of iSHPS cases, 21.5%, went for a second treatment for the concerned illness episode, significantly more than patients from the control sites. This may be due to several factors. First, since they mainly consulted health centres, quality of care at such facilities may be lower than at other facilities. However, a similar proportion of iSHPS cases, 22%, who initially consulted private providers went also for a second treatment. Another explanation may be that people residing in iSHPS areas have a better health literacy than those from control sites due to better health education programmes in that area and seek a second treatment when symptoms persist. It may also be that iSHPS patients were more able to afford a second treatment because they spent much less during initial treatment than their counterparts at control sites.

The majority of patients at all sites but CHEF went to private providers for their second treatment. At control sites, more than a third went to non-medical providers. Far fewer non-medical providers, 13%, were consulted at iSHPS sites, also during first treatment,15%, compared with a quarter of patients from CHEF sites and a third of patients at HoHEF sites. Many patients from iSHPS and CHEF sites who went for a second treatment did so at public providers, 40.3, and 36.4% respectively compared to 17.6% only for cases of HoHEF. In addition to the remark above, these figures suggest that inclusion of health centres in the HEF package may be necessary to stimulate health care seeking at all levels of public health providers.

Total cost for the second treatment ranged from US$6.1 to US$8.5 per patient and was cheapest at CHEF sites. Due to the relatively low proportion of patients who went for a second treatment at control sites combined with high total costs for the initial treatment, the incurred total costs of the second treatment did not contribute much to their overall costs for the concerned illness episode, about US$1.0. In contrast, total cost for the second treatment added 21% to the overall costs for patients at iSHPS sites.

Patients of the iSHPS incurred the lowest overall costs, US$10.4, 87 and 99% significantly lower than the amounts observed at CHEF and HoHEF sites respectively. Direct medical costs made up the largest part of these amounts, ranging from 85% at CHEF to 97% at HoHEF. The low transport expenses by the latter may suggest that their health seeking may also have been influenced by the cost of transport whereby they sought mainly care nearby as a cost saving measure [12].

The iSHPS arrangements clearly have most favourable results in terms of care seeking and OOPE for HEFB. The iSHPS emphasis on governance and quality improvement are in line with national policies, although its use of pay-for-performance and vouchers for underused services do add to programmatic costs. An economic evaluation using a provider and societal perspective of the iSHPS compared to stand-alone HEF would therefore add valuable information concerning the approach’s financial feasibility.

### Limitations

The study found interesting associations between the iSHPS approach, health seeking behaviour outcomes and associated costs, but it is not able to determine whether iSHPS caused these changes since it concerns a cross-sectional survey. A randomized cluster-controlled study, supported by qualitative research, would provide stronger evidence. Respondent characteristics across the sites were not homogeneous. For example, there were differences in age amongst those seeking care and children were more represented amongst the iSHPS sample, which may have positively influenced care seeking at public health providers. Distances to health providers for cases from CHEF sites differed from those at other sites, which may have influenced care seeking as Yanagisawa et al. [34] found significant less health centre consultations for poor people residing more than 2 km away from the facility. Another study using a 5 km cut-off found a similar influence on uptake of public health services [25]. However, distance did not appear to positively influence health centre utilisation in HoHEF areas as respective respondents lived on average significantly closer than those at the other sites. Other factors for which we did not control may have affected our observations. As such, we did not account for differences in case mix whereby disease patterns amongst concerned patients of the three groups may have differed. Health literacy may have been higher amongst the iSHPS households due to greater exposure to health related issues whereby they sought more care at public facilities [35]. This may be reflected by their low use of non-medical providers which is still relatively common in Cambodia and was especially pronounced at the control sites [36]. Financial literacy, the ability of an individual to make informed and effective decisions with their available financial resources [37], may have been better for iSHPS respondents so that they sought care from the cheapest provider. This may have gone hand in hand with perceived better quality of care and trust in public health providers [24], that both influence care seeking. Lastly, the degree of understanding of benefits of the HEF as well as the IDPoor card for eligible households may have affected the use of the HEF when sick. A multivariate regression analysis may clarify the influence of many of these confounding factors.

### Policy and research implications

To attract poor people to public health services under HEF arrangements in Cambodia there appears a need to concurrently optimize the supply- and demand side. Supply-side interventions should include those that stimulate more interactions between health care providers and the respective population to encourage relation building and foster trust and confidence. The HEF, as a social health protection scheme should ensure inclusion of health centres as frontline healthcare providers and promote them as the first point of contact with the public health system as it likely improves initiation of care seeking at such facilities, may increase overall utilization of public health facilities and reduces OOPE. Still, 44% of HEFB under iSHPS initiated care at private health providers, raising questions regarding the best interventions and configurations to stimulate care seeking at public health facilities.

Many issues remain that could be answered through appropriately designed studies. We did not assess nor decipher the extent of individual interventions on initiating care seeking at public health facilities, including pay-for-performance, vouchers, governance, or integration of HEF with voluntary insurance. We also did not identify the underlying reasons why such interventions, alone or in combination, influence care seeking. For example, is this due to better perceived quality of care or more confidence in providers? Therefore, it would be beneficial to ascertain the strength of each such intervention -alone or in combination- on care seeking behavior through quantitative studies and elicit the salient underlying influences. As each intervention has cost implications such assessments should be accompanied by an economic evaluation to see if the approach is financially feasible and sustainable. A considerable proportion of cases went for a second treatment for unknown reasons. Care seeking at non-medical providers at iSHPS sites was markedly lower than at the other sites.

## Conclusion

Arrangements to supplement HEF such as the iSHPS scheme under the SHPP that employ additional interventions like pay-for-performance, vouchers for underutilised services, quality improvement and focus on improved governance, appear to be better than stand-alone HEF in attracting sick HEFB to public health facilities and lowering their direct costs associated with health care seeking. Compared to other HEF arrangements, iSHPS saw 56% of HEFB initiate care seeking at public health facilities, much more than 13–40% at control sites. Inclusion of health centres in HEF arrangements appears instrumental to improve care seeking at all levels of public health facilities by HEFB. For unknown reasons, significantly more iSHPS cases went for a second treatment than at control sites. The overall costs associated with care seeking at iSHPS sites was US$10.4, which was 87 to 99% lower than control sites. Driving factors for these lower costs in comparison with control sites appeared to be the high use of primary health care facilities, lower user fees at public health facilities as well as at private facilities, and reduced tendency to seek care at non-medical providers.

## Abbreviations

CHEF: Comprehensive Health Equity Funds
HEF: Health Equity Funds
HEFB: Health Equity Fund Beneficiary
HoHEF: Hospital only Health Equity Funds
IDPoor: Identified poor
iSHPS: Integrated Social Health Protection Scheme
OD: Operational district
SHPP: Social Health Protection Program
